# Hashimoto’s Thyroiditis in children and adolescents: at presentation and during long-term follow up

**DOI:** 10.1186/1687-9856-2013-S1-P143

**Published:** 2013-10-03

**Authors:** You Jin Kim, Hae Sang Lee, Jin Soon Hwang

**Affiliations:** 1 Department of Pediatrics, Ajou University School of Medicine, Ajou University Hospital, Suwon, Korea

## Objective

Hashimoto’s thyroiditis (HT) is the most common cause of goiter and acquired hypothyroidism in children and adolescents. The aim of this study was to evaluate the clinical manifestations of HT leading to referral in children and adolescents, in addition to disease course and long-term outcome.

## Methods

The clinical and laboratory data of 57 patients with HT at presentation and long-term outcome were retrospectively evaluated using patient records.

## Results

The mean age of the patients at the time of diagnosis was 10.9 ± 2.3 years and female/male ratio was 49/8. The complaint at the time of hospital presentation was goiter in 66.7% of the patients. Other reasons for referral were clinical symptoms of hypothyroidism (7.1%) and findings on work-up for an unrelated problem (24.6%) or for high-risk groups (1.8%). At baseline, 49.1% (n=28) of the patients were euthyroid, whereas 31.6% (n=18) had subclinical hypothyroidism, 10.5% (n=6) of subjects were evaluated as hypothyroid. Out of 57 patients, 5 were diagnosed with hashitoxicosis. Five of the 28 subjects, who were initially euthyroid developed subclinical or overt hypothyroidism during the follow-up period and were started on thyroid medication.

## Conclusions

Thyroid function tests should be repeated periodically to detect progression to hypothyroidism in initially euthyroid patients as well as reversibility of hypothyroidism.

